# Clinical significance of MRI/^18^F-FDG PET fusion imaging of the spinal cord in patients with cervical compressive myelopathy

**DOI:** 10.1007/s00259-012-2192-y

**Published:** 2012-08-02

**Authors:** Kenzo Uchida, Hideaki Nakajima, Hidehiko Okazawa, Hirohiko Kimura, Takashi Kudo, Shuji Watanabe, Ai Yoshida, Hisatoshi Baba

**Affiliations:** 1 Department of Orthopaedics and Rehabilitation Medicine, Faculty of Medical Sciences, University of Fukui, Matsuoka Shimoaizuki 23, Eiheiji, Fukui 910-1193 Japan; 2 Department of Biomedical Imaging Research Center, University of Fukui, Matsuoka Shimoaizuki 23, Eiheiji, Fukui 910-1193 Japan; 3 Departments of Radiology, Faculty of Medical Sciences, University of Fukui, Matsuoka Shimoaizuki 23, Eiheiji, Fukui 910-1193 Japan; 4 Department of Radioisotope Medicine, Atomic Bomb Disease and Hibakusha Medicine Unit, Atomic Bomb Disease Institute, Nagasaki University, 1-12-4 Sakamoto, Nagasaki, 852-8523 Japan

**Keywords:** Cervical myelopathy, MRI, [^18^F]-Fluoro-deoxyglucose (FDG) positron emission tomography (PET), Fusion imaging, Spinal cord

## Abstract

**Purpose:**

^18^F-FDG PET is used to investigate the metabolic activity of neural tissue. MRI is used to visualize morphological changes, but the relationship between intramedullary signal changes and clinical outcome remains controversial. The present study was designed to evaluate the use of 3-D MRI/^18^F-FDG PET fusion imaging for defining intramedullary signal changes on MRI scans and local glucose metabolic rate measured on ^18^F-FDG PET scans in relation to clinical outcome and prognosis.

**Methods:**

We studied 24 patients undergoing decompressive surgery for cervical compressive myelopathy. All patients underwent 3-D MRI and ^18^F-FDG PET before surgery. Quantitative analysis of intramedullary signal changes on MRI scans included calculation of the signal intensity ratio (SIR) as the ratio between the increased lesional signal intensity and the signal intensity at the level of the C7/T1 disc. Using an Advantage workstation, the same slices of cervical 3-D MRI and ^18^F-FDG PET images were fused. On the fused images, the maximal count of the lesion was adopted as the standardized uptake value (SUV_max_). In a similar manner to SIR, the SUV ratio (SUVR) was also calculated. Neurological assessment was conducted using the Japanese Orthopedic Association (JOA) scoring system for cervical myelopathy.

**Results:**

The SIR on T1-weighted (T1-W) images, but not SIR on T2-W images, was significantly correlated with preoperative JOA score and postoperative neurological improvement. Lesion SUV_max_ was significantly correlated with SIR on T1-W images, but not with SIR on T2-W images, and also with postoperative neurological outcome. The SUVR correlated better than SIR on T1-W images and lesion SUV_max_ with neurological improvement. Longer symptom duration was correlated negatively with SIR on T1-W images, positively with SIR on T2-W images, and negatively with SUV_max_.

**Conclusion:**

Our results suggest that low-intensity signal on T1-W images, but not on T2-W images, is correlated with a poor postoperative neurological outcome. SUV_max_ of lesions showing increased signal intensity and SUVR measured on fusion MRI/PET scans are more sensitive parameters for predicting clinical outcome than signal intensity on the MRI scan.

## Introduction

It is important to assess spinal cord function in patients with cervical compressive myelopathy considered suitable for neurosurgical treatment. The majority of conventional tests focus on evaluation of neural conductivity across the damaged spinal cord [1], or morphological and pathological changes at the compressed cord that can be identified on MRI. MRI is valuable before surgical decompression because it visualizes not only the magnitude of the spinal cord compression but also the intramedullary signal intensity. Many authors have reported high intramedullary signal intensity on T2-weighted (T2-W) MR images in patients with compressive spondylotic lesions of the cervical spinal cord [2–5]. This abnormality in intramedullary signal intensity is considered to represent myelomalacia or cord gliosis secondary to longstanding compression of the spinal cord [4]. Therefore, the presence of high intramedullary signal intensity in patients with compressive myelopathy indicates the existence of a compressive spinal cord lesion of long duration. However, the prognostic capacity of these imaging parameters remains controversial, especially with regard to the change in signal intensity of the spinal cord on T2-W MR images. Increased signal intensity on T2-W MR images of the spinal cord and decreased signal intensity on T1-weighted (T1-W) images of the spinal cord are considered to predict a poor neurological outcome. The cause of controversy is thought to be the lack of quantitative assessment of these changes in signal intensity.

^18^F-FDG PET has been used to investigate the metabolic activity of neural tissue including the spinal cord [6]. We used high-resolution ^18^F-FDG PET to visualize the cervical spinal cord and quantify its metabolic activity [7], and have also found that patients with cervical myelopathy have a variable rate of glucose utilization in the whole cervical spinal cord [8], and that impaired glucose metabolic activity in these patients correlates closely with the severity of preoperative neurological dysfunction [9]. Recent studies by another group have demonstrated that regional changes in spinal cord ^18^F-FDG uptake have prognostic significance in cervical myelopathy [10, 11]. Thus, it is possible that the combination of MRI and ^18^F-FDG PET could uncover new features of cervical compressive myelopathy with respect to prognosis.

The present study was designed to evaluate the utility of 3-D MRI/^18^F-FDG PET fusion imaging in the detection of spinal cord lesions and to define intramedullary signal changes on MRI and local glucose metabolic rate measured on ^18^F-FDG PET scans in relation to clinical outcome and prognosis.

## Materials and methods

### Patient population

Between June 2008 and May 2011, 139 patients, 94 with cervical spondylotic myelopathy (CSM) and 45 with an ossified posterior longitudinal ligament (OPLL), underwent decompressive cervical spine surgery at our university medical centre. Of these 139 patients, 24 were enrolled in this study on the basis on the following criteria: (1) confirmation of monosegmental compression of the cervical spinal cord at the level of the spinal intervertebral disc in each patient (C2/3 in one patient, C3/4 in eight patients, C4/5 in seven patients, C5/6 in six patients, C6/7 in two patients) before surgery based on the presence of high intramedullary signal intensity on sagittal T2-W MR images obtained with a 1.5-T Signa system (General Electric, Milwaukee, WI); (2) lack of other lesions in the cervical vertebral column or spinal cord such as developmentally narrow canal detected on the plain radiograph, multisegmental lesions on MRI, or a history of traumatic cervical spinal cord injury; (3) lack of comorbidities such as diabetes mellitus; (4) providing signed consent.

The patients comprised 17 men and 7 women with a mean age at surgery of 64.5 years (range 34 to 86 years), and of these 24 patients 20 had CSM and 4 had OPLL. The mean duration of neurological symptoms was 14.8 months (range 3–36 months). Patients with a high intramedullary signal intensity underwent both 3-D MRI for a second time and ^18^F-FDG PET for this clinical study during the week before surgery. Anterior decompression with autogenous iliac bone grafting was performed in 20 patients, and en bloc open-door laminoplasty was performed in 4 patients. All examinations including the ^18^F-FDG PET study strictly followed the Ethics Review Committee Guidelines of Fukui University and written informed consent was obtained from all patients. The ^18^F-FDG PET study was undertaken as an Advanced Medical Technology Development Project at Fukui University.

Neurological assessment was conducted in accordance with the Japanese Orthopedic Association (JOA) scoring system for cervical myelopathy. The mean period of neurological follow-up was 14.1 months (range 6–36 months). The rate of neurological improvement was calculated by the following equation: (postoperative JOA score − preoperative JOA score)/(17 − preoperative JOA score) × 100. An improvement of 100 % was the best possible postoperative recovery [12].

### High-resolution MRI

MRI examination of the spinal cord for the MRI/PET fusion study was performed preoperatively using 3.0-T Signa system (General Electric). T1-W and T2-W sagittal images of the spinal cord were obtained using a fast spin echo sequence. An additional 3-D fast spoiled gradient echo sequence was used for T1-W imaging with contiguous thin sections of 0.4 mm covering the entire cervical spine region. Quantitative analysis was conducted as described previously [13]. T2-W midsagittal images of the cervical spinal cord with increased signal intensity were obtained first because small changes in signal intensity on T1-W images are difficult to visualize, and the region of interest (ROI) is only 0.05 cm^2^. The same spinal cord lesions identified on T1-W and T2-W images were viewed on a Hurry PACS (Cosmo Medical Systems Co., Osaka, Japan) with ROIs of 0.05 cm^2^. T2- and T1-W midsagittal MR images of the cervical spinal cord with normal signal intensity (level of the C7/T1 disc) with ROI 0.3 cm^2^ were also obtained. The signal intensity was measured on the PACS client viewer, and the differences in signal intensity in the cord were qualitatively assessed on both T1-W and T2-W images. In quantitative analysis of the differences in signal intensity, the signal intensity ratios (SIR) on both T1-W and T2-W images were calculated as the ratio between the increased lesional signal intensity in the midsagittal plane (ROI 0.05 cm^2^) and the signal intensity in the midsagittal plane at the level of the C7/T1 disc (ROI 0.3 cm^2^).

### ^18^F-FDG PET study

The ^18^F-FDG PET examination of the cervical spinal cord was performed preoperatively using a GE Advance system (General Electric). This system allows simultaneous acquisition of 35 transverse slices with an interslice spacing of 4.25 mm with septa (2-D mode). The PET camera was cross-calibrated using a cylindrical phantom filled with ^18^F^−^ solution. Images were reconstructed to a full-width at half-maximum of 4.2 mm in both transaxial and axial directions. The field of view and pixel size of the reconstructed images were 256 mm and 2 mm, respectively. Subjects were studied after fasting for at least 4 h. Transmission scans were obtained over 10 min using a standard ^68^Ge/^68^Ga rod source for attenuation correction of the emission images. ^18^F-FDG was injected at a dose of 185 MBq into an antecubital vein over 10 s. Static scans were then obtained 50 min after injection over 10 min.

### MRI/^18^F-FDG PET image fusion and assessment

Using an Advantage workstation (GE Healthcare), the same slices of the cervical 3-D MRI scan obtained using a 3.0-T Signa system and the ^18^F-FDG PET images were fused automatically and manually by identifying the cerebellar tonsil and laryngopharynx as the corresponding landmarks. To determine the glucose metabolic rate of the cervical spinal cord, round ROIs each 4 mm in diameter were placed on the spinal cord on the same lesions identified on the sagittal fused MR images. The reconstructed tissue activity images were converted to standardized uptake value (SUV) images corrected for the injected dose and patient body weight using the following equation: SUV = tissue activity in kilobecquerels per millilitre/(injected ^18^F-FDG dose in megabecquerels/body weight in kilograms).

The ROI counts were determined in lesions showing increased signal intensity in the sagittal plane and at the level of the C7/T1 disc. For the same lesion, the maximal ROI count (SUV_max_) was then adopted as the tissue radioactivity to reduce the partial volume effect [14, 15]. Furthermore, at the level of the C7/T1 disc, the mean ROI count (SUV_mean_) was adopted to reduce the individual differences.

In a similar manner to SIR, the SUV ratios (SUVR) were calculated as the ratios between the SUV_max_ of the cervical spinal cord lesions showing increased signal intensity on fused MR images in the sagittal plane (diameter 4 mm) and the SUVmean of the normal cervical spinal cord at the level of the C7/T1 disc (diameter 4 mm).

### Data analysis

Pearson partial correlation analysis was used to examine the relationship among neurological scores, SIR, SUV_max_, SUVR and duration of the disease. All statistical analyses were conducted using the Statistical Package for Social Sciences (version 15.0; SPSS, Chicago, IL).

## Results

**Fig. 1:**
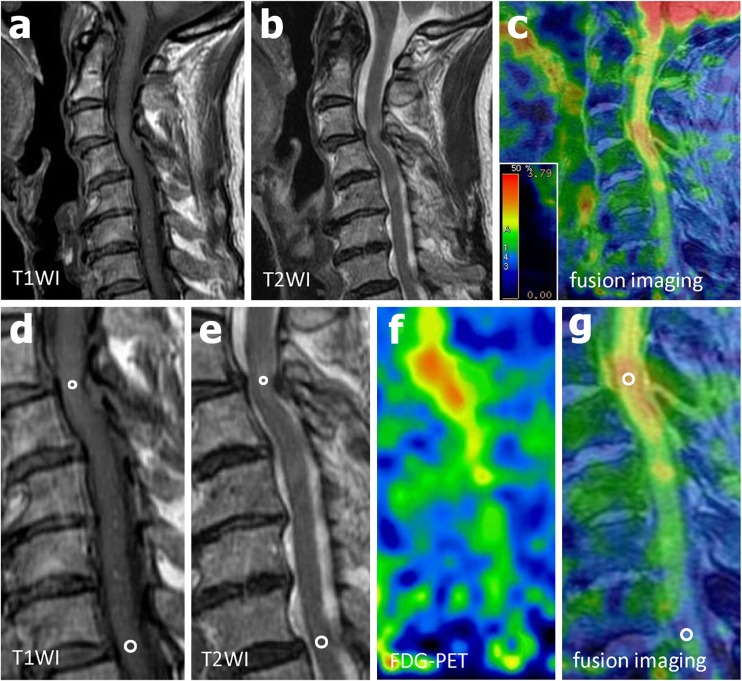
Patient 22. An 80-year-old woman with cervical spondylotic myelopathy was treated surgically and showed a neurological improvement rate of 75.0 % at follow-up. **a** Midsagittal T1-W MR image. **b** Midsagittal T2-W MR image. **c** MRI/PET fusion image. **d** T1-W MR image for calculation of SIR as the ratio of the signal intensity in the midsagittal area of increased signal intensity (*small circle*) to the signal intensity in the midsagittal area of normal signal intensity at the level of the C7/T1 disc (*larger circle*), **e** T2-W image for calculation of SIR in a similar manner. **f** Sagittal ^18^F-FDG PET image. **g** Sagittal MRI/PET fusion image demonstrates a focal increase in ^18^F-FDG uptake at the level of a lesion showing increased signal intensity. SUVR is calculated as the ratio between the SUV_max_ of the lesion (*circle* diameter 4 mm) and the SUVmean of the normal cord at the level of the C7/T1 disc (*circle* diameter 4 mm)

**Fig. 2:**
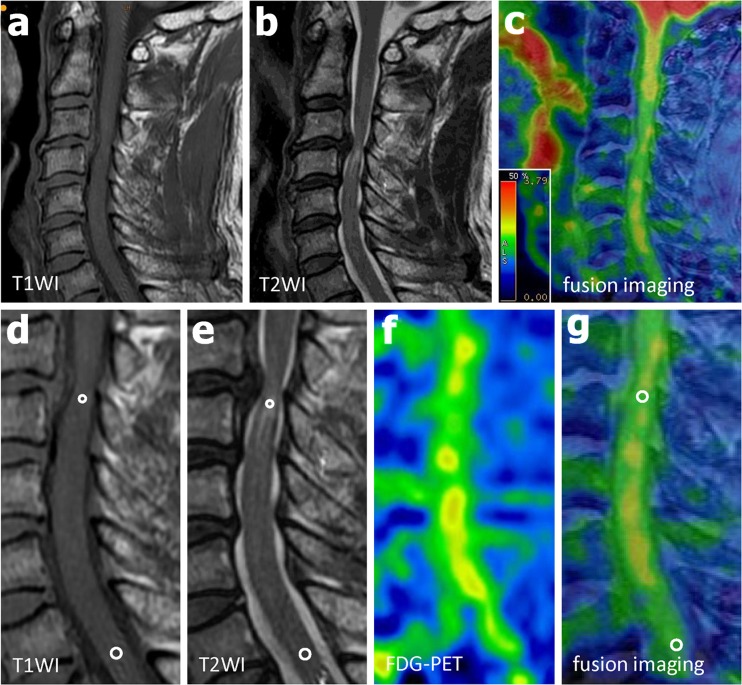
Patient 16. A 62-year-old man with cervical spondylotic myelopathy was treated surgically and showed a poor neurological improvement rate of 28.6 % at follow-up. **a** Midsagittal T1-W MR image. **b** Midsagittal T2-W MR image. **c** MRI/PET fusion image. **d** T1-W MR image for calculation of SIR as the ratio of the signal intensity in the midsagittal area of increased signal intensity (*small circle*) to the signal intensity in the midsagittal area of normal signal intensity at the level of the C7/T1 disc (*larger circle*), **e** T2-W image for calculation of SIR in a similar manner. **f** Sagittal ^18^F-FDG PET image. **g** Sagittal MRI/PET fusion image demonstrates inconspicuous ^18^F-FDG uptake at the level of a lesion showing increased signal intensity. SUVR is calculated as the ratio between the SUV_max_ of the lesion (*circle* diameter 4 mm) and the SUVmean of the normal cord at the level of the C7/T1 disc (*circle* diameter 4 mm)

Table 1 summarizes the demographic data of the patients. Correlation analysis showed no significant correlation between age and SUV_max_ (*R* = 0.127). The SUV_max_ for the lesions showing increased signal intensity in the sagittal plane on T2-W images showed various patterns (1.60–2.89). Examples are shown in Figs. 1 and 2. Figure 1 (patient 22) shows a significant increase in ^18^F-FDG uptake (SUV_max_ 2.50, SUVR 1.84) at the level of the lesion (SIR on T1-W image 1.29, SIR on T2-W image 1.55). The rate of neurological improvement was considered good (75.0 %) at follow-up. On the other hand, Fig. 2 (patient 16) demonstrates no increase in ^18^F-FDG uptake (SUV_max_ 1.60, SUVR 0.87) at the level of the lesion (SIR on T1-W image 0.99, SIR on T2-W image 1.48). The rate of neurological improvement at follow-up was poor (28.6 %).

**Table 1:** Demographic data of the patients

Patient	Age (years)	Sex	Disease	Affected level^a^	Surgical procedure	Duration (months)	JOA scores		Improvement rate (%)	SIR		SUV_max_	SUVR
							Preoperative	Postoperative		T1-W images	T2-W images		
1	54	F	CSM	4/5	Ant	12	14	16	66.7	1.13	1.90	2.78	1.55
2	77	F	CSM	3/4	Ant	12	13	15	50.0	1.09	1.16	2.29	1.19
3	51	M	CSM	3/4	Ant	30	8	10	22.2	0.80	2.47	1.81	0.86
4	59	F	CSM	4/5	Ant	20	12	15	60.0	1.06	1.13	2.37	1.30
5	68	M	OPLL	5/6	Ant	36	10	15	71.4	0.92	1.82	1.89	1.15
6	57	M	CSM	3/4	Ant	20	12	16	80.0	1.15	1.63	2.56	1.82
7	62	F	CSM	4/5	Ant	10	11	16	83.3	1.24	1.45	2.70	1.90
8	45	M	CSM	5/6	Ant	18	11	14	50.0	1.03	1.05	2.24	1.17
9	71	M	OPLL	5/6	Post	14	14	16	66.7	1.04	1.54	2.21	1.40
10	64	M	CSM	4/5	Ant	12	11	16	83.3	1.02	1.59	2.45	1.74
11	74	F	CSM	3/4	Ant	8	11	14	50.0	1.19	1.97	2.44	1.24
12	86	F	OPLL	5/6	Ant	10	10	14	57.1	1.02	1.08	2.21	1.18
13	74	M	CSM	5/6	Ant	20	13	16	75.0	1.11	1.55	2.17	1.48
14	76	M	CSM	4/5	Ant	4	12	15	60.0	1.07	1.30	2.39	1.40
15	80	M	CSM	3/4	Post	4	10	15	71.4	1.16	1.14	2.42	1.59
16	62	M	CSM	6/7	Ant	30	10	12	28.6	0.99	1.48	1.60	0.87
17	68	M	CSM	5/6	Ant	12	11	15	66.7	1.19	1.54	2.35	1.31
18	70	M	CSM	3/4	Ant	18	13	16	75.0	1.10	1.38	1.89	1.50
19	34	M	CSM	2/3	Post	14	11	16	83.3	1.23	1.34	2.89	1.58
20	57	M	CSM	4/5	Ant	18	13	15	50.0	1.04	1.84	2.07	1.06
21	44	M	CSM	4/5	Ant	10	13	16	75.0	1.28	1.50	2.43	1.50
22	80	F	CSM	3/4	Ant	3	13	16	75.0	1.29	1.55	2.50	1.84
23	61	M	CSM	6/7	Ant	18	11	15	66.7	1.20	1.26	2.90	1.93
24	74	M	OPLL	3/4	Post	2	13	13 → 15	50.0	1.09	1.27	2.30	1.21

*CSM* cervical spondylotic myelopathy, *OPLL* ossified posterior longitudinal ligament, *Ant* anterior decompression, *Post* en bloc open-door C3-7 laminoplasty.

^a^Site of maximal compression of the cervical spinal cord.

### Relationship between SIR on MRI and clinical outcome

**Fig. 3:**
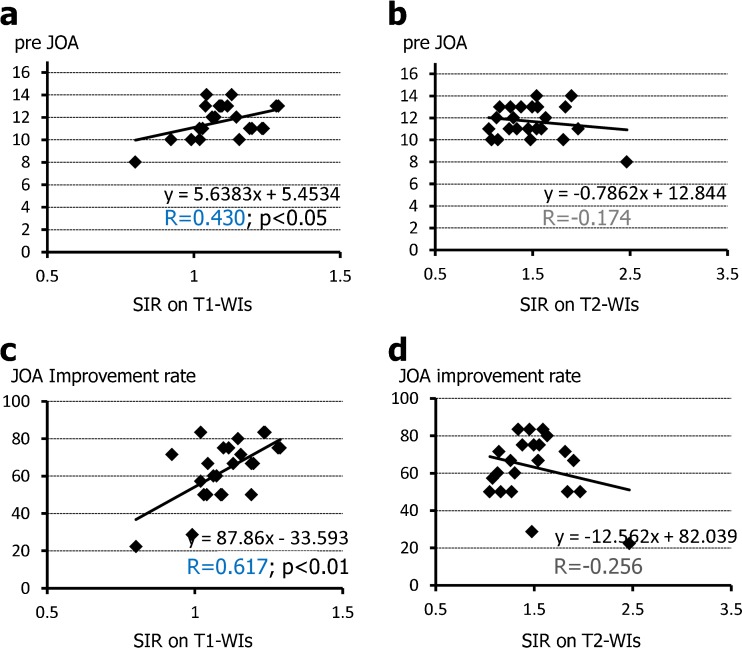
Relationship between SIR and neurological scores. Preoperative JOA scores (**a**, **b** *pre JOA*) and postoperative neurological improvement (**c**, **d** *JOA improvement rate*) are significantly correlated with SIR on T1-W images (**a**, **c**) but not on T2-W images (**b**, **d**). The values of *R* in *blue* indicate weak correlations, the values in *black* indicate no correlation

Figure 3 shows the relationship between SIR on T1-W images and T2-W images and neurological scores. The SIR on T1-W images was correlated significantly with the preoperative JOA score (*R* = 0.430; *p* < 0.05; Fig. 3a) and postoperative neurological improvement (*R* = 0.617; *p* < 0.01; Fig. 3c). However, there were no significant correlation between the intramedullary signal intensity on T2-W images and the preoperative JOA score (*R* = −0.174; Fig. 3b) or postoperative neurological improvement (*R* = −0.256; Fig. 3d).

### Clinical significance of SUV_max_ for lesions showing increased signal intensity

**Fig. 4:**
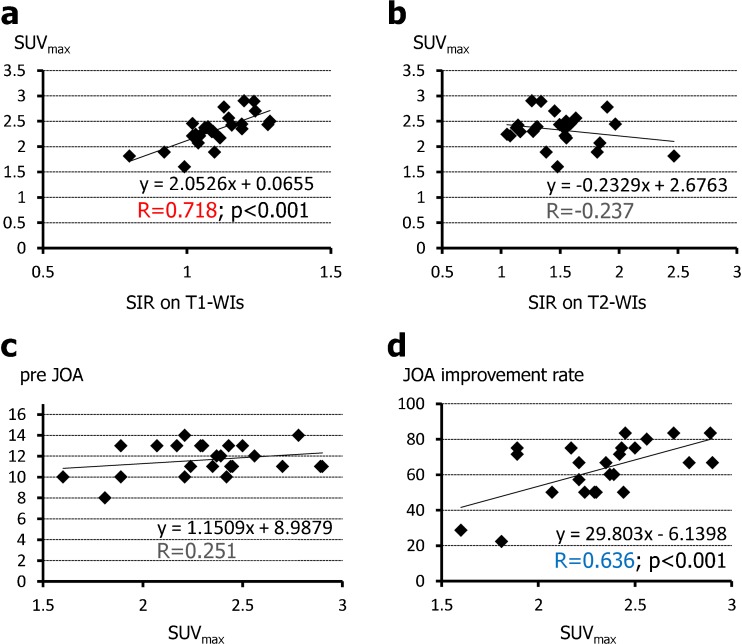
Relationship between SUV for the lesions and signal intensity on MRI/PET fusion images and SIR and neurological scores. Lesional SUV_max_ values are strongly correlated with SIR on T1-W images (**a**) but not on T2-W images (**b**). SUV_max_ values are not significantly correlated with preoperative JOA scores (**c** *pre JOA*), but are significantly correlated with postoperative neurological improvement (**d** *JOA improvement rate*). The value of *R* in *red* indicates a strong correlation, the value in *blue* indicates a weak correlation, the values in *black* indicate no correlation

Figure 4 shows the relationship between SUV_max_ for lesions showing increased signal intensity in the sagittal plane and SIR on T1-W and T2-W images as well as neurological scores. SUV_max_ for the lesions was correlated significantly with SIR on T1-W images (*R* = 0.718, *p* < 0.001; Fig. 4a) but not on T2-W images (*R* = −0.237; Fig. 4b). Furthermore, SUV_max_ was correlated with postoperative neurological improvement (*R* = 0.636, *p* < 0.001; Fig. 4d) but not with the preoperative JOA score (*R* = 0.251; Fig. 4c).

### Clinical significance of the SUV ratio (SUVR)

**Fig. 5:**
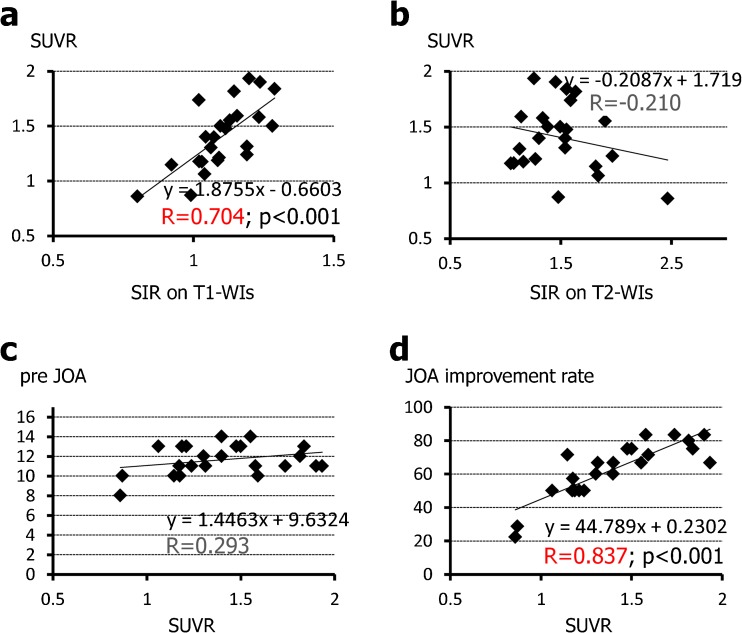
Relationship between SUVR on MRI/PET fusion images and SIR and neurological scores. SUVR was strongly correlated with SIR on T1-W images (**a**) but not on T2-W images (**b**). SUVR was not significantly correlated with preoperative JOA scores (**c** *pre JOA*), but was significantly correlated with postoperative neurological improvement (**d** *JOA improvement rate*). The values of *R* in *red* indicate a strong correlation, the values in *black* indicate no correlation

Figure 5 shows the relationship between SUVR and SIR on T1-W images and T2 -W images as well as neurological score. SUVR was correlated significantly with SIR on T1-W images (*R* = 0.704, *p* < 0.001; Fig. 5a) but not on T2-W images (*R* = −0.210; Fig. 5b). Furthermore, SUV_max_ was correlated significantly with postoperative neurological improvement, and the correlation coefficient was much higher than that for SUV_max_ for lesions showing increased signal intensity on the sagittal plane (*R* = 0.837, *p* < 0.001; Fig. 5d), but not with preoperative JOA score (*R* = 0.293; Fig. 5c).

### Effect of disease duration on SIR and SUV_max_ for lesions showing increased signal intensity

SIR on T1-W images and T2-W images and SUV_max_ for lesions showing increased signal intensity were correlated with the duration of symptoms of myelopathy. A longer duration of symptoms was associated with a lower SIR on T1-W images (*R* = −0.616, *p* < 0.01), but with a higher SIR on T2-W images (*R* = 0.421, *p* < 0.05). Furthermore, SUV_max_ was correlated negatively with duration of symptoms (*R* = −0.453, *p* < 0.05).

## Discussion

Several groups have investigated the factors involved in the prognosis in patients with CSM and OPLL [5, 12, 16–18]. Recognition of the functional capacity and functional normalization of the chronically damaged spinal cord are important clinical issues. It is also important to know those factors that determine neurological improvement after surgery. Age at surgery [16], duration of neurological symptoms [5, 16, 17, 19–21], and existence of signal changes on preoperative MRI [2–4, 22, 23] have been considered key predictors of surgical outcome. These factors could also have had a significant impact on the surgical outcome in the patients in the present study as well, although we focused on changes in the signal intensity on MRI and local glucose metabolism in monosegmental spinal cord lesions on PET using high-resolution MRI/PET fusion imaging.

While MRI provides the highest specificity in the assessment of morphological changes and the intramedullary state of the spinal cord, it is almost impossible to estimate the potential recovery of the spinal cord on preoperative MRI without quantitative analysis. Furthermore, the signal intensities on MRI scans are irregular because different sequence parameters are set for each individual patient. On the other hand, ^18^F-FDG PET allows visualization of the metabolic activity of the spinal cord neural tissue under the same conditions in each scan, and this parameter correlates closely with neurological prognosis. We have previously quantified glucose utilization in the cervical spinal cord of patients with myelopathy [8]. Furthermore, Kamoto et al. [7] reported that the normal metabolic rate of glucose utilization in the cervical spinal cord (SUV) in healthy Japanese subjects (aged 40–70 years) was 1.93 ± 0.23, although the value was found to be 2.12 ± 0.48 by Nakamoto et al. [24] and 1.84 ± 0.23 by Floeth et al. [11]. In another study, we found that patients with mild to moderate myelopathy have a high SUV of the entire cervical spinal cord, while those with marked and profound tetraparesis had a low SUV [9]. Thus, a high SUV seems to reflect hyperactive neuronal activity within the spinal cord. In our latest study, patients with poor neurological improvement rate were found to have low preoperative SUV and a low level of postoperative neurological improvement [25]. Unfortunately, our results showed that preoperative SUV was not significantly different among patients with increased signal intensity on MRI. In reconsidering the above findings, we believe that the major problems in such analysis are (1) the lack of clear grouping of T1-W and T2-W images on MRI, (2) the use of SUV values that represented the average of the entire cervical spinal cord, rather than local SUV, as applied in the present study, and (3) the difficulty in identifying lesions showing an increase in signal intensity by PET.

The MRI/PET fused images were constructed in the present study based on preliminary studies and our previous experience. The 3-T MRI scanner has an in-plane resolution capability of 400 μm and any slice can be shown in 3-D by MRI at 3 T—a feature hitherto unavailable with the 1.5-T MRI system. The combination of the same slice imaged with the two highest-end modalities in near real-time on simultaneous scans has enabled us to image metabolic function with detectable accuracy at the level of the cervical spinal cord. In this study, we were able to assess accurately and in detail the metabolic activity of lesions showing increased signal intensity. Our results showed that ^18^F-FDG PET is more suitable and sensitive for the detection of changes in low signal intensity on T1-W images on MRI/PET fusion imaging. Our results also suggested that SUV_max_ for lesions showing increased signal intensity correlates with SIR on T1-W images and postoperative neurological outcome, but not with SIR on T2-W images. Additionally, the SUVR predicted neurological improvement more sensitively than SIR on T1-W images or SUV_max_ in lesions showing increased signal intensity. Floeth et al. [10] found a significant decrease in ^18^F-FDG uptake in the area of the lower cervical cord in patients with myelopathy than in the control group. This could be the reason that made SUVR a more sensitive predictor than SUV_max_ in lesions showing increased signal intensity.

A high signal intensity on T2-W MR images indicates local pathological changes in the spinal cord, and patients with compressive myelopathy with high signal intensity on T2-W MR images usually have a poorer prognosis even after surgical intervention [4, 22]. Recent studies have indicated that qualitative or semiquantitative analysis of MR images is a simple and easy technique in the clinical setting [13, 26–29]. In contrast, there is no significant correlation between high signal intensity on T2-W MR images and postoperative prognosis, and statistical analysis has also failed to confirm this correlation [5, 23, 30]. The present quantitative study based on MRI/PET fusion images also showed no correlation with SIR on T2-W images, while SIR on T1-W images was correlated with preoperative JOA score and postoperative neurological improvement. High signal intensity is observed on T2-W MR images in patients with early stage myelomalacia. Some patients at an intermediate stage display a variable degree of cystic necrosis of the central grey matter, which is better visualized on T2-W MR images. Finally, central cystic degeneration, syrinx formation and atrophy are the main features of late-stage myelomalacia [31–33]. On the other hand, several groups have confirmed that low signal intensity on preoperative T1-W MR images is a predictor of a poor prognosis [23, 30, 34]. Based on the results reported in the literature, high intensity signal on T2-W images at early stages of compressive myelopathy are indicative of oedema and gliosis (which may be reversible), whereas low intensity signal on T1-W images could reflect myelomalacia and necrosis (which are considered irreversible). Decompression of the spinal cord by surgery can restore blood circulation in the spinal cord, reduce swelling, and enhance regeneration of nerve fibres, thus reducing or normalizing the high signal intensity in the intramedullary region. In the late stage, necrosis and syrinx become irreversible, making surgery ineffective.

Changes in ^18^F-FDG uptake at the site of the compressed spinal cord seem to reflect its neuronal activity at different stages of the pathophysiological course of myelopathy. Spinal cord autopsy findings in patients with late-stage cervical myelopathy include demyelination, white matter axonal loss and grey matter neuronal loss [35, 36]. In contrast, Yamamoto et al. [37] found the presence of highly active neurogenic progenitor cells in the grey matter in the adult rat spinal cord, and that these cells could differentiate into neurons and glial cells following mechanical stimulation. In the *twy*/*twy* mouse model (a model of human OPLL) our group found altered immunoreactivity to neurotrophins in the compressed spinal cord. The early stages of cord compression were associated with increased immunoreactivity of brain-derived neurotropic factor and neurotrophin-3 in neurons and astroglial cells in the grey matter, which may explain the increased glucose metabolism [38]. In the early stages, the process is still reversible and a good clinical outcome after surgical intervention is expected. Long-term compression, however, leads to atrophy and necrosis of the anterior grey horn cells and the loss of glucose-consuming neurons and glia cells, resulting in decreased ^18^F-FDG uptake in the spinal cord during the late stages. On the other hand, Esik et al. [39] have shown increased ^18^F-FDG utilization in patients with myelitis, together with an increased number of sodium channels along the demyelinated segments of injured axons. The observed changes in glucose metabolism on ^18^F-FDG PET in cervical myelopathy could reflect neuronal plasticity in the compressed spinal cord, neural activity, deterioration of synaptic function, loss of neurons and neural tissue, changes in blood flow, and the magnitude of mechanical compression. Although the present study was limited by the small sample size, we believe that glucose metabolism (SUV) measured on MRI/PET fusion images is suitable for visualization of the metabolic activity of neural tissue, and that ^18^F-FDG PET is a more sensitive tool for assessment of the clinical outcome and prognosis in patients with cervical compressive myelopathy.

### Conclusion

This is the first study measuring regional glucose metabolism in the affected spinal cord of patients with cervical compressive myelopathy using MRI/PET fusion imaging. 3-D MRI/^18^F-FDG PET fusion imaging is useful for mapping the exact level of a cervical spinal cord lesion on the ^18^F-FDG PET image. Assessment of neurological function by ^18^F-FDG PET may be equal to or better than that assessed in term of signal intensity on T1-W images. Preoperative glucose utilization rate on ^18^F-FDG PET imaging seems suitable for prediction of postoperative neurological outcome.
